# PrionHome: A Database of Prions and Other Sequences Relevant to Prion Phenomena

**DOI:** 10.1371/journal.pone.0031785

**Published:** 2012-02-20

**Authors:** Djamel Harbi, Marimuthu Parthiban, Deena M. A. Gendoo, Sepehr Ehsani, Manish Kumar, Gerold Schmitt-Ulms, Ramanathan Sowdhamini, Paul M. Harrison

**Affiliations:** 1 Department of Biology, McGill University, Montreal, Quebec, Canada; 2 Department of Biophysics, University of Delhi (South Campus), New Delhi, India; 3 National Centre for Biological Sciences, GKVK Campus, Bangalore, India; 4 Tanz Centre for Research in Neurodegenerative Diseases, and Department of Laboratory Medicine and Pathobiology, University of Toronto, Toronto, Ontario, Canada; 5 Department of Biosciences, Biochemistry, Åbo Akademi University, Turku, Finland; INSERM, UMR-S747, France

## Abstract

Prions are units of propagation of an altered state of a protein or proteins; prions can propagate from organism to organism, through cooption of other protein copies. Prions contain no necessary nucleic acids, and are important both as both pathogenic agents, and as a potential force in epigenetic phenomena. The original prions were derived from a misfolded form of the mammalian Prion Protein PrP. Infection by these prions causes neurodegenerative diseases. Other prions cause non-Mendelian inheritance in budding yeast, and sometimes act as diseases of yeast. We report the bioinformatic construction of the PrionHome, a database of >2000 prion-related sequences. The data was collated from various public and private resources and filtered for redundancy. The data was then processed according to a transparent classification system of prionogenic sequences (*i.e.*, sequences that can make prions), prionoids (*i.e.*, proteins that propagate like prions between individual cells), and other prion-related phenomena. There are eight PrionHome classifications for sequences. The first four classifications are derived from experimental observations: prionogenic sequences, prionoids, other prion-related phenomena, and prion interactors. The second four classifications are derived from sequence analysis: orthologs, paralogs, pseudogenes, and candidate-prionogenic sequences. Database entries list: supporting information for PrionHome classifications, prion-determinant areas (where relevant), and disordered and compositionally-biased regions. Also included are literature references for the PrionHome classifications, transcripts and genomic coordinates, and structural data (including comparative models made for the PrionHome from manually curated alignments). We provide database usage examples for both vertebrate and fungal prion contexts. Using the database data, we have performed a detailed analysis of the compositional biases in known budding-yeast prionogenic sequences, showing that the only abundant bias pattern is for asparagine bias with subsidiary serine bias. We anticipate that this database will be a useful experimental aid and reference resource. It is freely available at: http://libaio.biol.mcgill.ca/prion.

## Introduction

Prions are alternative, propagating states of normal cellular proteins, that propagate from organism to organism, through infection or inheritance. Prions were originally defined as the causative agent of mammalian transmissible spongiform encephalopathies (TSEs), diseases which include scrapie in sheep and Creutzfeldt-Jakob disease (CJD) in humans [1]. CJD involves progressive dementia, and death within a year of diagnosis. TSEs can arise in inherited, sporadic or infectious forms. Infectious prions lack nucleic acids [1], and rely on the presence of a host prion-protein gene for propagation [2]. Whereas the normal cellular form of the prion protein (PrP-C) is mostly alpha-helical [3] [4], the infectious form of the prion protein (PrP-Sc), is mostly beta-sheet [5], which indicates a dramatic conformational change in the infectious protein.

The prion protein (PrP) is highly conserved across mammals, typically with >50% sequence identity relative to human PrP [6] [7]. PrP maintains very high sequence conservation (>95%) in regions associated with disease, and also conserves a metal ion–binding repetitive region whose copy number is implicated in some human prion diseases [8], and which is intrinsically disordered when not bound to metal ions [9]. Mammalian PrP paralogs have continued to be discovered and have been demonstrated to be of neurological relevance and functionally linked to PrP [10]. Doppel is a divergent PrP homolog (∼25% sequence identity), that is mainly expressed in the testis, and can cause neurodegeneration when aberrantly expressed in the central nervous system (CNS) [10]. A second paralog of PrP, dubbed Shadoo, is more highly conserved across vertebrates, than either Doppel or PrP. Shadoo intriguingly shares a high degree of sequence identity with PrP in the short alanine-rich stretch that forms a transmembrane alpha-helix in some disease-associated PrP products [11]. Shadoo is expressed in the CNS, and both Shadoo and PrP-C can counteract Doppel neurotoxicity in a similar way [12]. Furthermore, down-regulation of Shadoo indicates a pre-clinical event in the response to prion infection [13]. PrP homologs have also been observed in fish [14], and extensive genome-scale analyses in vertebrates have led to the discovery of additional gene and pseudogene family members for PrP [15]. Moreover, distant homology to ectodomains in the ZIP family of proteins linked to cytosolic divalent metal ion import, indicates that the PrP gene family may have originated from an ancestral ZIP sequence in an early common metazoan ancestor [16].

Apart from the original infectious prion phenomena just described, prions have been defined in other organisms. In the budding yeast *S. cerevisiae*, prions have been identified as cytoplasmic or nuclear elements inherited in a non-Mendelian fashion [17] [18] [19]. The first two yeast prions discovered were [*PSI^+^*] and [URE3] [17] [18] [19]. [*PSI^+^*] arises from propagation of a misfolded amyloid form of Sup35p, part of the translation termination complex. Formation of [*PSI^+^*] prions reduces the efficiency of translation termination and increases levels of nonsense-codon readthrough [18]. Such readthrough has been demonstrated to be a potential mechanism to uncover cryptic genetic variation [20] [21]. [URE3], the prion form of the nitrogen catabolism protein Ure2p, functions to up-regulate poor nitrogen source usage, even when rich sources are available [17]. Prions may also be considered as diseases of budding yeast, in certain contexts [22] [23]. A defining characteristic of the known yeast prions is a region with an obvious bias for asparagine (N) and/or glutamine (Q) residues [24] [25] [26] [27]. Mutation of these residues reduces the ability of proteins to add onto wild-type prion aggregates [26] [27]. Indeed, randomization of the sequences of prion-determinant domains for [*PSI^+^*] and [URE3] does not block prion formation [28] [29], and prion formation propensity in N/Q-rich domains can be predicted with reasonable accuracy solely using amino-acid composition [30]. These biases are linked to protein disorder; the prion determinant regions of both Ure2p and Sup35p are intrinsically disordered in their native forms [31] [32]. Previously, we have shown that these N/Q biases are maintained in fungi that are estimated to have diverged from each other ∼1 billion years ago; furthermore, there is evidence for purifying selection, to varying degrees, on different prion domains or subdomains [33]. Several hundred prion-like domains (with pronounced bias for N, Q and other subsidiary biases) occur in the proteomes of diverse fungi and higher eukaryotes [24] [33] [34]. N/Q-rich domains also form aggregates in other contexts; the protein CPEB from the sea-slug *Aplysia* has a prion-like domain which can behave like a prion in budding yeast, which also forms aggregates within *Aplysia* neuronal cells, and which may function in long-term memory formation [35] [36]. Other N/Q-rich domains are implicated in human diseases and form aggregates (*e.g.* TDP-43 and FUS [37]). Recent work indicates that N residues tend to be more enriched in prionogenic domains, and that Q bias (such as that observed in the Sup35p prion determinant) is less usual [38].

The universe of prion phenomena continues to expand in budding yeast [38] [39] [40] [41] [42], and in other fungi, *i.e,* the Het-S prion in *Podospora anserina* [43]. For example, in budding yeast, a prion that is formed by the Cyc8 protein is part of a transcriptional regulatory complex that regulates 7% of yeast genes, thus potentially regulating their expression *en masse* [44]. Although the vast majority of described prions arise *via* propagation of alternative amyloid states of proteins, other types of prions are possible, such as the [ß] prion, which arises through propagation of the auto-activated state of yeast protease B [45]. Also, some amyloids, dubbed ‘prionoids’, exhibit evidence of cell-to-cell propagation of aggregates in some contexts or experimental conditions, and may demonstrate limited organism-to-organism propagation in experiments [46]. For example, the prionoid SOD1, whose amyloid formation is implicated in amyotrophic lateral sclerosis, has been shown to propagate from cell to cell in a neuro-2a cell system [47]. Other prion-related phenomena, which involve propagation of aggregates of prion-related domains within cells, or within model systems (*e.g.*, CPEB, FUS or TDP-43 mentioned above), are also increasingly being reported and utilized for research into the contribution of aggregate formation to disease and protein function.

Here, we report the bioinformatic derivation of a classification system for prion–related sequences, which is used for the computational construction of a public-domain resource, the PrionHome database. This database enables tracking of the rapidly growing corpus of prion phenomena and prion-related sequence data. The PrionHome comprises collated and curated information about prions, prionogenic sequences, ‘prionoids’, prion protein orthologs and paralogs, prion-related pseudogenes (*i.e.*, genes copies that appear to have lost their protein-coding ability), prion protein interactors and candidate-prionogenic sequences predicted from compositional analysis. The database entries contain information about prion determinants, compositionally-biased and disordered regions, protein interactors, protein structures, genomic coordinates, key citations, and comments supporting the classification of the sequences in the database. We demonstrate the utility of the database in examining relationships between prion-related molecules, through sequence compositional analysis of the database data.

## Materials and Methods

### Database structure

**Figure 1:**
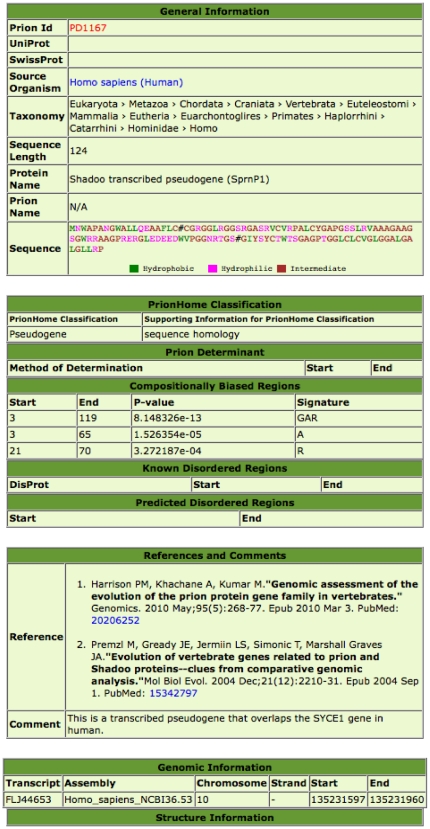
A screenshot of an entry in the PrionHome database, for the Shadoo transcribed pseudogene in the human genome.

The PrionHome database is a record-based database, similar in format to the DisProt database [48]. It is managed and updated using the SQL and PHP scripting languages. The individual database entries are for single sequences. A screen-shot example is depicted in Figure 1. This is the database entry for a duplicated transcribed pseudogene in the human genome that is homologous to Sprn, the gene encoding the Shadoo protein. Database entries are indexed by Prion Identifier (which is in the form PDxxxx, where xxxx is a four-digit number), and also by PrionHome Classification (which is explained below in the section describing the database entry format).

### Overview of data collation and curation

**Figure 2:**
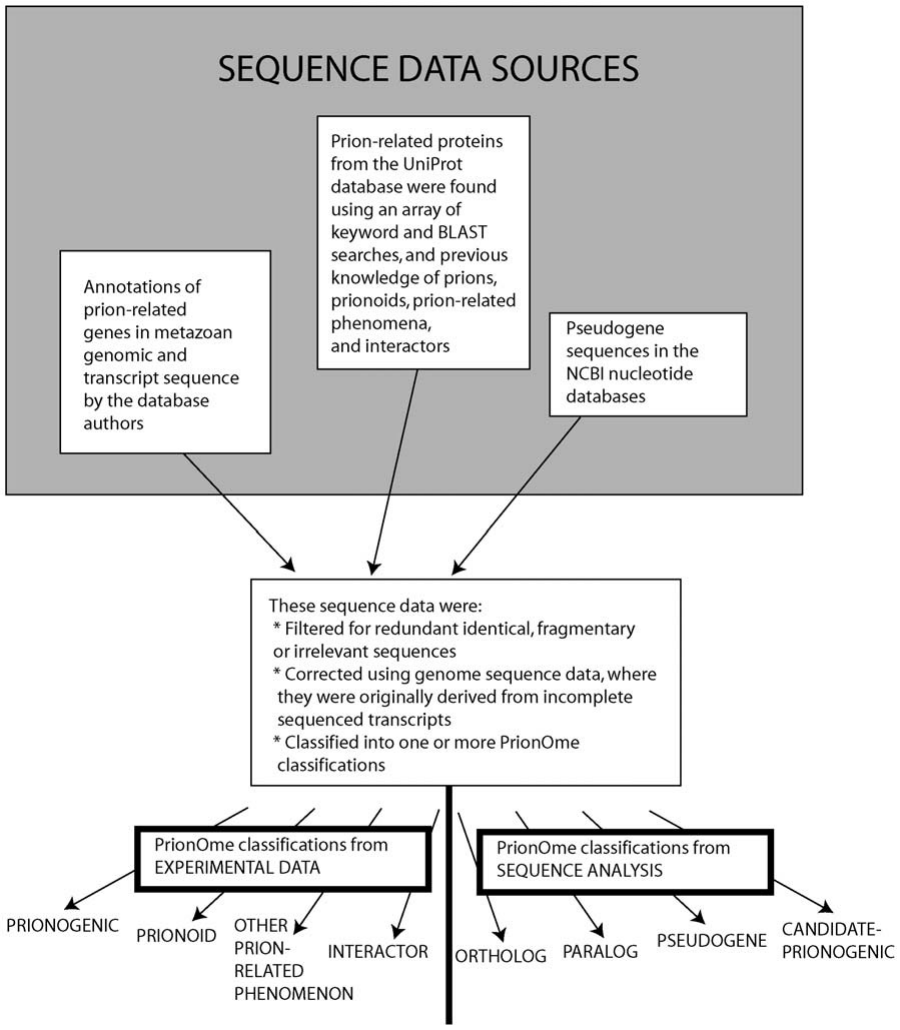
A flow-chart showing a summary of the data collation and curation.

The sequence data is derived from several sources (Figure 2). The first source is a set of annotations (made by the authors) of Prion Protein Family relatives, including pseudogenes, and distant homologs not detectable by standard sequence alignment [15] [16]. Also, protein sequences were extracted from the UniProt database using keyword searches for relevance to prion phenomena, using sequence homology searches to known prion-related protein sequences, and also using our previous knowledge of prions, prionoids, and other prion-related protein sequences. These were filtered appropriately for redundancy, or sometimes updated using genomic sequence data where they were originally derived from translations of incomplete transcripts. Furthermore, some additional sequences for pseudogenes were taken from NCBI nucleotide sequence databases.

To put the sequences into their appropriate PrionHome classification categories, a variety of data sources were applied. Information about prions, prionoids and other prion-related phenomena was collated from the literature. Protein interaction data was extracted from the EBI IntAct database [49]. Orthologs and paralogs were calculated using simple sequence comparison with BLAST [50], where they had not already been inferred in previous investigations (for more details, see the description of PrionHome classifications in the Database Entry Format section below). Candidate-prionogenic sequences were refined from previous annotations of ‘prion-like’ domains by the authors [33] [51], and also are taken from predictions made using an Hidden Markov Model (HMM) algorithm by Alberti, *et al*. [38]. More specific details of the data collation and curation, where relevant, are listed below in the database entry format section, along with descriptions of the data sources and methods used for annotations within the database entries.

### Database Entry Format

There are nine sections for each database entry. Further details about database entry format can be found on the help page of the database. The database entry sections are as follows:

### (i) General Information

This first section contains general identifying information: a PrionHome database ID in the form PDxxxx, where xxxx is a four-digit number; UniProt identifiers (some PrionHome entries do not have such identifiers, *e.g.*, new genome annotations for Prion Protein homologs or pseudogenes); source organism and taxonomy information; sequence length; protein name; the complete protein sequence, and the *prion name*, where relevant.

The *prion name* field only applies to ‘prionogenic’ sequences, *i.e.*, sequences that can make prions. The name of the prion phenomenon for which the sequence is prionogenic is indicated. Prion names follow the standard convention for prions in fungal genetics [52]. The original pathogenic prion in mammals has been given the name *[PrP TSE prion]*. Entries labeled ‘Alberti, *et al* (2009) data’, are proteins from the analysis of Alberti, *et al.* [38], which were indicated as likely prions via a combination of fluorescence microscopy, SDD-AGE analysis, Sup35C heritable-switch prion assay and in-vitro assembly assay [38]. In addition, for each prion name, the *prion type* is given. Prions are classified into two types, as follows:

Type Am: The prion state of the protein is a an alternative conformational amyloid isoform.

Type Ac: The prion state is an activated state of an enzyme, or other protein or protein complex.

### (ii) PrionHome Classification

This section of the database entry contains the PrionHome classification, as well as a short description of the supporting information for this classification. The total numbers of entries with each PrionHome Classification is summarized in Table 1. The PrionHome classification system consists firstly of four classifications derived from experimental data, and secondly four derived from sequence analysis. The PrionHome Classification can be one or more of these listed below:

**Table 1:** Summary of database content.

PrionHome Classification	Number ofDatabase Entries
Prionogenic	51
Prionoid	6
Other prion-related phenomenon	10
Interactor	460
Ortholog	958
Paralog	205
Candidate-Prionogenic	411
Pseudogene	13
TOTAL*	2003

*This is not an arithmetic sum of the individual PrionHome Classification categories, since some database entries have multiple PrionHome Classifications.

**Figure 3:**
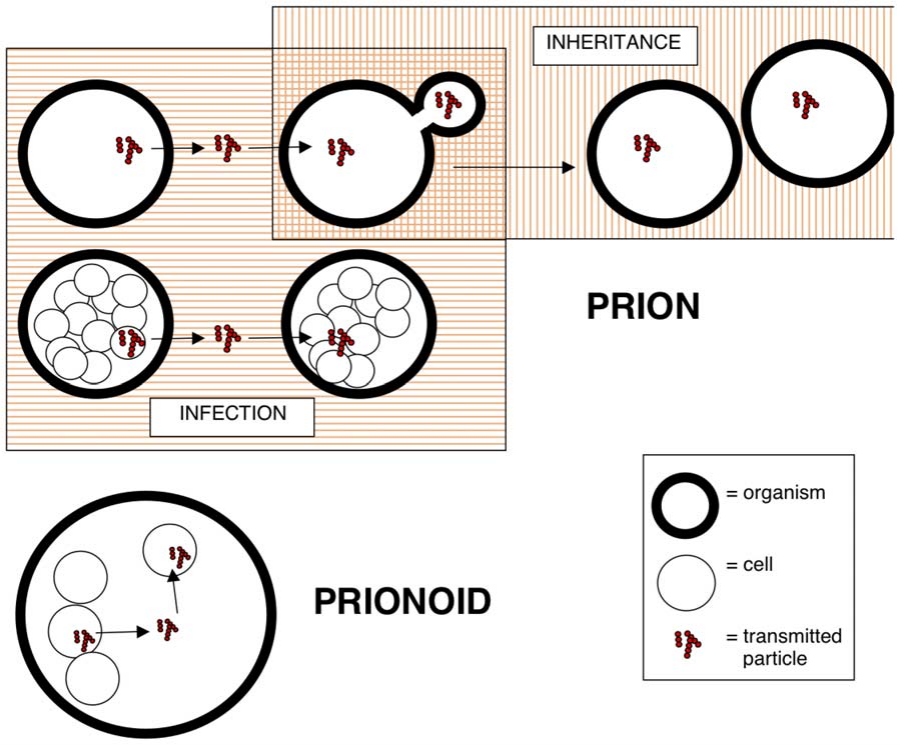
A graphical depiction of how transmission differs for *prions* and *prionoids*, as defined in the database. A key that explains the symbols in the figure is included.

*Prionogenic:* A protein sequence that is known to form prions. These were collated by the database authors from the literature. Currently here, we define a prion as any unit of propagation of an altered state of a protein or proteins; the prions are made from an endogenous protein, expressed in the cells of an individual organism; they are either propagated by infection into other individual organisms, or by inheritance to progeny organisms. A graphical depiction of this definition of prion transmission is shown in Figure 3.

*Prionoid:* A protein with a altered conformational state, which demonstrates cell-to-cell propagation, in the absence of evidence for natural organism-to-organism infectivity (Figure 3). The definition of the term ‘prionoids’ is coined and discussed in the review Aguzzi and Rajendran [46]. Here, we restrict our definition to proteins from multicellular organisms. Some prionoids demonstrate experimental organism-to-organism transmission.

*Other prion-related phenomenon:* A protein that has some behaviour like a prion, but does not fit the definition of prion or prionoid. For example, the MAVS protein forms aggregates that propagate intracellularly in response to viral infection; also, MAVS aggregation can be ‘seeded’ by introduction of aggregates previously made *in vitro* [53].

*Interactor:* Proteins shown to interact with a prionogenic protein. Data in this first release of the PrionHome database are restricted to entries listed as binary interactors of prionogenic proteins, taken from the IntAct database [49].

*Ortholog:* An ortholog of a sequence that is known to form prions (*i.e.*, of prionogenic sequences). Orthologs are the mutually most similar sequences in different organisms. These were calculated as orthologs using the bi-directional best hits approach. Some orthologous proteins are from novel genome annotations, annotated in Harrison, *et al.* (2010) [15]. Orthologs for yeast prionogenic sequences are only calculated for complete fungal proteomes. Orthologs of the Het-S prion-determinant domain were detected via remote homolog detection techniques, and modelled in 3D by protein threading [54].

*Paralog:* A paralog of a sequence that is known to form prions. Paralogs are duplications of protein sequences within the same genome. Some orthologous proteins are from novel genome annotations, annotated in Harrison, et al. (2010) [15]. ZIP metal-ion import proteins that contain a Prion Protein -related domain, that were detected through sensitive Hidden Markov Model analysis [16], are classified as paralogs in the PrionHome database.

*Pseudogene:* A copy of a prion-related gene that is formed via retrotransposition, or other processes of duplication, followed by coding-sequence disablement [55]. These annotations were taken from Harrison, et al. (2010) [15], and other literature.

*Candidate-Prionogenic Sequence:* A protein sequence that contains a domain that is a candidate for being prionogenic, but that has not been shown to be prionogenic experimentally. Candidate Prionogenics have two categories:

- *N/Q-biased:* These have domains with an obvious bias for glutamine and/or asparagine residues, with optional contributing biases for glycine, serine and tyrosine, and biases against charged and hydrophobic residues, as observed in the first four described yeast prions. These were determined using the binomial probability minimization algorithm described in the ref. [51], with a maximum binomial P-value of 1×10^−10^. These Candidate-Prionogenic proteins are biased purely for N and/or Q residues, or for N and/or Q residues with a subsidiary compositional bias for Y, S or G (with P-value<1×10^−4^), and do not have contributing biases from charged residues {DERK} or major hydrophobic residues {VILM}, with P-value<1×10^−4^. These criteria were derived from analysis of the four first yeast prion determinants that were determined (Sup35p, Ure2p, Rnq1p and New1p). All of the amyloid-based (*i.e.*, Type Am) prionogenic sequences in budding yeast have domains with N/Q bias.
- *Alberti-HMM:* These are predicted by the HMM algorithm used in Alberti, *et al.* [38], trained on the first four described yeast prion determinants (in Sup35p, Ure2p, Rnq1p and New1p).

These definitions of Candidate-Prionogenic refer exclusively to prions from budding yeast, and not to those from *P. anserina* or from mammals. The latter prions are made largely from hydrophobic domains. The Alberti-HMM data set contains 35 further Candidate-Prionogenic sequences that were not covered by the data set derived from analysis of N/Q bias and other compositional biases [51]. The two methods used to define candidate prionogenic sequences are thus complementary in providing greater coverage for detection of possible prionogenic cases. The HMM algorithm is trained specifically on four prion determinants, and may miss cases that look less like these four determinants, but which may be detected using more general compositional principles defined using the bias probability-minimization algorithm.

The categories of ‘N/Q-biased’ or ‘Alberti-HMM’ for Candidate-Prionogenic sequences are listed in brackets after the PrionHome classification designation in each database entry. Sequences in both categories are labelled ‘N/Q-biased, Alberti-HMM’.

### (iii) Prion Determinant

This is the part of the protein sequence that forms the prion determinant, *i.e.*, that is required for prionogenic activity. In this section, the method of determination for the prion determinant (curated from the literature), and its start and end points in the sequence, are indicated. In some cases the prion determinant is determined algorithmically [38] [51].

### (iv) Compositionally-Biased (CB) Regions

These are regions of the protein sequence that have a bias towards a subset of amino-acid residue types. They are defined using the algorithm described in refs. [24] [51] [56]. In this section are indicated the following: the start and end points in the sequence; the binomial P-value for the CB region; the ‘signature’ of the compositionally-biased region. In the signature are listed the one-letter codes of amino-acid residue types that define the biased region, in decreasing order of importance [51]. That is, ‘NY’ indicates a region that is rich in N and Y. All compositional biases with binomial P-value< = 10^−7^ are listed.

### (v) Known Disordered & (vi) Predicted Disordered Regions

Known disordered regions are taken from the DisProt database [48]. The ID from the DisProt database, and the start and end positions of the disordered region in the protein sequence are listed. Other disordered regions are predicted using the DISOPRED algorithm, and default parameter values [57]. The start and end positions of the predicted disordered region in the protein sequence are listed.

### (vii) References and Comments

References were curated from the literature. Original references are given that describe the demonstration of a prionogenic determinant, or, if not appropriate, for the sequencing of a prionogenic sequence, or ortholog or paralog. Additional references are provided if the sequence has been updated, or if additional significant sequence annotations are derived. Original references for the determination of interactors are also given. Comments on the other sections of the database entry are recorded in this section. Specifically also, for any sequence database entry, are listed the PrionHome Database identifiers (PDxxxx) of other sequences in the database, for which that sequence is an interactor, paralog or ortholog. For example, if PD8888 is an interactor of the prionogenic sequence PD7777, then the term, ‘Interacts with PD7777’ is listed in the Comment section of the entry for PD8888, and *vice versa*. The terms ‘Ortholog of PDxxxx’ and ‘Paralog of PDxxxx’ are used in a similar way.

### (viii) Genomic Information

In this section, we list the transcripts IDs (taken from the EMBL database); and the coordinates of the gene of the protein sequence in a recent genome assembly. These genome coordinate annotations are taken from the work in Harrison, et al. (2010) [15], or otherwise transferred from the Ensembl database [58]. Listed are the name of the genome assembly, the chromosome, strand direction and start and end points of each exon.

### (ix) Structure Information

Coordinates of structures for the PrionHome database entries are listed here. These are either experimental structures, or comparative models. Experimental structures are taken from the PDB [59]. They are colour-coded according to the source of the data (Red for NMR spectroscopy; Green for X-ray crystal structure; Magenta for EM (Electron Microscopy); and Blue for a comparative model). Comparative models were made using the program MODELLER [60] for PrP-related proteins for which the sequence alignment cannot be made automatically. These proteins occur in fish species, and tend to have large, interspersed disordered loops.

## Results and Discussion

### User Interface

**Figure 4:**
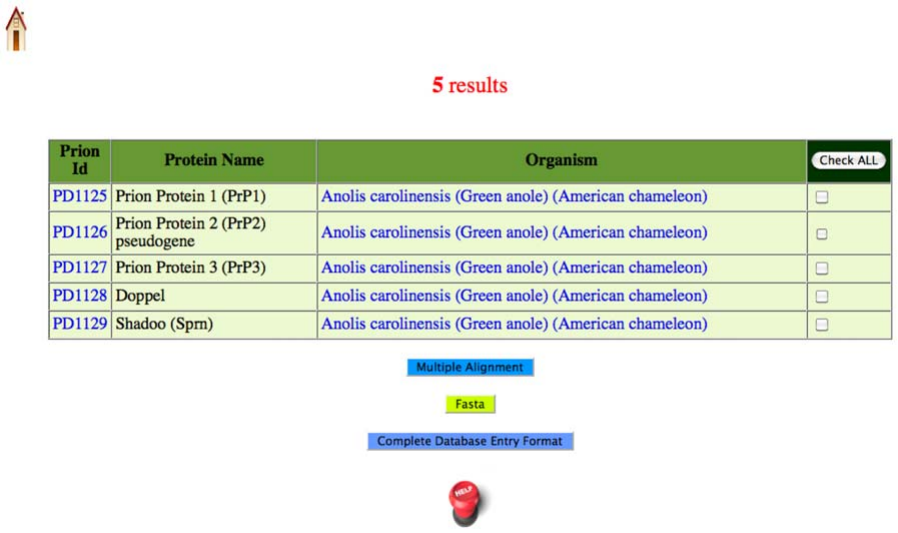
An example of the results of a keyword search, for the term ‘Anolis’ in the ‘Organism’ field. Five entries for *Anolis caroliensis* (a lizard), appear listed.

The user interface is designed so that investigators can select and download subsets of prion-related sequence and annotation data, that are of interest. A comprehensive ‘Help’ page is provided. The database can be accessed by:

- *Browsing:* A ‘Browse’ link on the home page enables users to browse the complete listing of PrionHome database entries and select any entries that they want. The selected entries can then be downloaded in complete database entry format, FASTA format (*i.e.*, just the protein sequence), or as a CLUSTALW multiple sequence alignment [61], using the labeled buttons on the bottom of the display page.
- *Searching:* Users can ‘Search’ either by keyword, by SQL query, or by BLAST interface. For keyword search, the database or field in which to search must be selected from a pull-down menu. For searching the sequences in the database, users can specify a regular expression of the sort used by the ELM database of linear motifs [62]. A screenshot of a keyword search result for the phrase *Anolis* (a lizard) in the ‘Organism’ field is shown in Figure 4. Database entries can be downloaded in one of three formats (complete database entry format, FASTA format or CLUSTALW multiple sequence alignment), as detailed above for ‘Browsing’. For searching the database using the BLAST query box, the user can either paste in a single sequence in FASTA format, or upload one from a file, and then specify the parameters of the BLAST search.

In addition, on the home page, there are links to convenient lists of database entries, grouped by: *(i)* Prion Name, *(ii)* PrionHome Classification, *(iii)* Organism, and *(iv)* Supporting Information for PrionHome Classification. In some cases, the database entries belong to more than one PrionHome Classification. For example, there are eleven database entries that are classified as both ‘Paralog’ and ‘Interactor’. There are separate links for these database entries that have multiple PrionHome Classifications. A ‘PrionHome News’ window is also provided, in which updates and changes to the PrionHome database are documented.

### Examples of Database Usage

We anticipate that the database will be useful as a reference resource for prion biologists, since it enables access to curated, up-to-date listings of a variety of prion-related sequences. In addition, the database will be useful to investigators as a bioinformatic aid to experimental design. Database entries contain cross-references to other database entries (that are interactors or homologs), and to relevant entries in other databases (*e.g.*, DisProt [48]).

Database entry browsing can be informative for experimental design. For example, examination of the interactors of Ure2p, the protein that forms the [URE3] prion, produces some interesting results. The entry accession for Ure2p, the protein that forms the [URE3] prion, is PD0040. As instructed in the Help page, to find PD0040 interactors ,we perform a keyword search in the ‘Comment’ field for ‘Interacts with PD0040’. By doing this, we find a list of 70 proteins that interact with Ure2p. Browsing through these, we find several that are also candidate prions according to the survey of Alberti, *et al.* [38], *e.g.*, the PrionHome database entry PD0734. This is the G-protein-coupled receptor GPR1, which was shown, *via* the dihydrofolate reductase reconstruction technique, to interact with Ure2p [63]. The database entry indicates that the GPR1 protein contains a long (>60 amino acid residues) region that is compositionally-biased for asparagine, similar to the prion determinant in Ure2p. The fragment of the GPR1 protein that has prion-like composition has been shown to efficiently induce [URE3] prion formation [43]. This protein can thus work as a prion ‘cross-seeder’, a potentially promiscuous mechanism through which prions can act as inducers of each other [43] [64]. Another interactor and potential cross-seeder of Ure2p found in the database is MSN1 (PD0729), which encodes a candidate-prionogenic domain of ∼100 residues long.

In Table 2, we have presented some data analysis of prion-related sequences resulting from queries to the PrionHome database, made using the SQL query interface. Firstly, for Table 2, we queried the PrionHome database for mammalian sequences of the Prion Protein (PrP) family, that have the sequence motif ‘YYR’. This motif is an epitope for an antibody that is specific for the disease-related prion form of the PrP protein, PrP^Sc^ [65]. We find that all of the PrP sequences that have so far been shown to be prionogenic, retain this YYR sequence motif, as well as the substantial majority of other sequences orthologous to PrP (Table 2). However, none of the paralogous sequences contain this tripeptide (Table 2).

**Table 2:** Counts of mammalian sequences in the PrionHome database with the sequence motif tyrosine-tyrosine-arginine (YYR).

PrionHome Classification	# of sequences with the YYR sequence motif	# of sequences without the YYR sequence motif
Prionogenic PrP sequences (Major Prion Protein)	20	0
Orthologs of Major Prion Protein	413†	9
Paralogs of Major Prion Protein	0	120

† As an example, the SQL query to obtain this data is: *SELECT prion_id , name FROM main WHERE sequence REGEXP ‘YYR’ AND prion_type = ‘Ortholog’ AND taxonomy LIKE ‘%mammal%’.*

In Table 3, we investigated the composition of prion-related domains in *Saccharomyces cerevisiae* (budding yeast). Known amyloid-type (Type *Am*) prionogenic sequences in *S. cerevisiae* tend to have a pronounced bias for asparagine and/or glutamine residues [33] [51]. Subsidiary biases (*e.g.*, for aromatic residues or serines and threonines) may also be important for honing prion propagation mechanisms. A microcrystal structure of a small peptide fragment of the Sup35p prion determinant domain indicated that prion domains can be stabilized by hydrogen bonds between Q and N sidechains [66]. Also, sidechains that contain six-membered rings, such as phenylalanine (F) and tyrosine (Y) may be able to interact in an arrangement termed *π* stacking [67]. Tyrosine residues in glutamine-rich domains improve the fragmentation of amyloid fibrils made from these domains, leading to more efficient amyloid propagation in the absence of Rnq1/PIN or sometimes Hsp104 [68]. Toombs, *et al.* showed that phenylalanine, tryptophan and tyrosine residues have very high prion formation propensities [30]. In [*PSI^+^*] prions, aromatic side-chain interactions outside of the amyloid core cause oligomerization that leads to formation of prion strain conformations with more limited amyloid cores [69]. We checked how many of the prionogenic and candidate-prionogenic sequences from budding yeast in the PrionHome database also have a compositional bias for F or Y residues (Table 3). A substantial fraction of the domains classed as prionogenic contain an F/Y compositional bias (6/25, 24%). Also, a smaller fraction of candidate-prionogenic domains (as defined in *Database Construction & Content*), contain an F/Y compositional bias (90/377, 13%). Thus, these domains could use *π* stacking for amyloid stabilization, in addition to asparagine and glutamine sidechain hydrogen-bonding interactions; also, they could also have increased fibril fragmentation, in cases with the highest F/Y biases [68]. There is a depletion for this compositional feature for a set of candidate-prionogenic sequences that were experimentally shown not to form amyloids (Table 3) [38]. These data thus suggest that a subsidiary F/Y bias has a generally positive effect on prionogenesis.

**Table 3:** A summary of prionogenic (Amyloid Prion Type Am) and candidate-prionogenic sequences in the PrionHome database from *Saccharomyces cerevisiae,* that have N/Q and F/Y compositional biases.

PrionHome Classification	# of sequences that have N/Q bias, but no F/Y bias	# of sequences that have N/Q bias, and F/Y bias*
Prionogenic**	19	6***
Candidate-Prionogenic ****	329	48
Candidate-Prionogenic experimentally shown not to form amyloid*****	16	2

*F/Y compositional biases with binomial P-values< = 10^−6^ are considered (see *Database Construction & Content* for details).

**This data includes the Alberti, et al. (2009) data from screens for candidate prionogenic sequences.

***As an example, the SQL query to obtain this data is: *SELECT prion_id , name FROM main WHERE prion_type LIKE’%prionogenic%’ AND bias REGEXP ‘Y|F’ AND bias REGEXP ‘N|Q’ AND organism LIKE ‘%cerevisiae%’.*

****Polymorphic sequences have been removed from these totals. This is the total list of Prion-Like sequences from Harrison, et al. (2006) and Alberti, et al. (2009).

*****Prion-like sequences that failed to form amyloid by any tests in Alberti, et al. (2009).

To probe further the compositional details of the budding-yeast amyloid-type prionogenic sequences, we have performed a case-by-case statistical analysis of the main and subsidiary biases in each sequence (Table 4). The biases for each sequence were calculated as described in the section on *Database Construction & Content*, using the lowest-probability subsequence algorithm [51]. Of the eight well-characterized prionogenic sequences, five have a predominant N bias ([NU+], [ISP+], [MOT3+], [SWI+] and [URE3]), and three a predominant Q bias ([RNQ+], [PSI+], [OCT+]). Adding in the seventeen further prionogenic sequences from ref. [38], gives us a total of 18/25 with predominant N bias, and 7/25 with predominant Q bias. *Aplysia* CPEB is also Q-rich and behaves as a prion in yeast cells [35] [36]. Thus all of the known amyloid-type prionogenic sequences have a predominant N or Q bias, but there is an obvious general preference for Ns over Qs. Recent experimental analysis indicates that N residues promote formation of propagatable amyloid, while Qs induce formation of other non-prion conformers. Halfmann *et al.* substituted Qs in prionogenic sequences with Ns and *vice versa* [70]. Prionogenic sequences with Qs replaced by Ns are likely to form prions as well, whereas prionogenic sequences with Ns substituted for Qs do not form prions [70]. These data suggest that the encoding of prionogenesis in Q-rich sequences is more dependent on specific subsidiary biases and sequence patterns, than in N-rich prionogenic sequences. Interestingly, here, subsidiary N bias occurs in four of the seven prionogenic sequences that have a main Q bias.

**Table 4:** Detailed analysis of compositional biases in budding yeast amyloid-type Prionogenic sequences.

Database Identifier	Name and Prion [in square brackets]	Compositional biases*
PD0023	Serine/threonine-protein_kinase_CBK1[Alberti, et al. 2009 data]	188/249/5.4e-46/Q; 85/179/2.2e-12/SNP
PD0026	Prion_formation_protein_1_NEW1 [NU+]	68/94/8.5e-27/NY; 60/103/3.6e-10/Y; 332/417/4.7e-08/SLV
PD0028	Asparagine-rich_protein_NRP1 [Alberti, et al. 2009 data]	391/567/4.7e-56/N; 383/715/1.6e-48/**NS**
PD0029	SWI/SNF_chromatin-remodeling_complex_subunit_SWI1 [SWI+]	4/322/3.2e-67/N 336/384/1.1e-36/Q; 913/1260/1.6e-20/**NS**I; 14/31/8.2e-09/T; 87/298/9.1e-09/S; 592/692/1.0e-08/K; 568/585/1.3e-08/N
PD0033	U6_snRNA-associated_Sm-like_protein_Lsm4 [Alberti, et al. 2009 data]	93/166/6.5e-26/N
PD0034	Uncharacterized_protein_YBL081W [Alberti, et al. 2009 data]	30/353/2.3e-48/**NS**YHQ; 195/353/1.9e-21/S; 48/153/1.1e-07/Y
PD0035	Nuclear_and_cytoplasmic_polyadenylated_RNA-binding_protein_PUB1 [Alberti, et al. 2009 data]	242/287/4.7e-26/NM; 419/452/1.5e-21/Q; 273/303/8.4e-11/M
PD0040	Protein_URE2 [URE3]	2/78/3.2e-25/N
PD0044	Nitrogen_regulatory_protein_GLN3 [Alberti, et al. 2009 data]	142/630/4.0e-59/**NS**; 124/617/2.4e-30/N; 146/630/2.7e-26/S; 644/716/1.7e-10/SN; 68/119/3.1e-08/TD
PD0734	G_protein-coupled_receptor_GPR1 [Alberti, et al. 2009 data]	490/557/2.1e-59/N; 91/288/1.7e-14/IFNYW; 675/736/2.9e-12/KWY; 854/947/4.1e-07/SN
PD0920	Mediator_of_RNA_polymerase_II_transcription_subunit_3_PGD1 [Alberti, et al. 2009 data]	277/373/7.6e-29/QNM; 256/392/7.1e-14/N; 202/259/2.2e-08/AP
PD0921	Uncharacterized_RNA-binding_protein_YPL184C [Alberti, et al. 2009 data]	5/27/2.7e-27/N; 98/124/1.2e-10/Q
PD2094	global_transcriptional_regulator_Sfp1 [ISP+]	21/517/3.2e-34/**NS**HIT; 531/554/7.4e-23/D; 3/20/1.34e-07/T; 9/502/2.5e-07/S; 311/327/3.6e-07/Q; 139/155/4.9e-07/A; 213/517/3.4e-05/H; 175/202/9.9e-05/Y; 467/490/1.1e-04/D; 556/570/2.1e-04/N; 614/628/4.4e-04/H; 26/136/5.8e-04/I; 510/525/6.4e-03/S
PD0021	General_transcriptional_corepressor_CYC8 [OCT+]	492/586/2.2e-81/QA; 698/952/7.5e-30/ESTPNAQ; 14/29/2.9e-23/Q; 509/555/4.6e-14/A; 116/373/2.7e-08/YW
PD0022	Uncharacterized_protein_YBR016W [Alberti, et al. 2009 data]	40/100/6.2e-33/QYN; 5/96/1.0e-07/Y
PD0024	Eukaryotic_peptide_chain_release_factor_GTP-binding_subunit_SUP35 [PSI+]	4/122/1.8e-49/QYNG; 158/221/3.7e-16/EK; 12/112/2.3e-11/Y; 138/218/7.9e-10/K; 138/218/7.9e-10/K; 4/108/1.1e-08/N
PD0025	RNQ1 [RNQ+]/[PIN+]	123/402/5.6e-82/QNSGY; 185/402/6.5e-16/N; 7/344/7.1e-13/S; 50/381/1.3e-10/G
PD0027	mRNA-binding_protein_PUF2 [Alberti, et al. 2009 data]	909/1062/2.5e-51/N; 52/486/3.2e-31/SQTNP; 241/470/4.7e-10/Q; 710/756/5.8e-09/LTIN; 854/1015/2.8e-08/SQ; 55/254/6.2e-08/T; 140/476/8.5e-08/N
PD0031	Zinc_finger_protein_YPR022C [Alberti, et al. 2009 data]	158/319/8.1e-42/Q; 20/472/1.2e-15/NPS; 732/1040/2.8e-09/NI
PD0032	transcription_factor_RLM1 [Alberti, et al. 2009 data]	212/629/4.5e-55/**NS**P; 228/629/3.4e-18/S; 94/353/3.1e-10/N; 186/549/8.8e-10/P; 451/475/1.5e-08/Q; 119/133/2.6e-07/D
PD0036	transcriptional_activator/repressor_MOT3 [MOT3+]	4/488/4.4e-45/NH; 7/34/3.0e-26/Q; 231/337/2.1e-10/P; 4/396/1.4e-09/H; 431/449/2.6e-07/AS
PD0037	Serine/threonine-protein_kinase_KSP1 [Alberti, et al. 2009 data]	538/939/4.7e-33/**NS**H; 53/83/1.3e-10/D; 590/972/1.5e-08/S; 454/494/9.6e-08/K
PD0038	Nucleoporin_ASM4 [Alberti, et al. 2009 data]	52/496/5.7e-25/**NS**; 18/48/4.8e-15/Q; 52/426/2.4e-07/S
PD0043	Nucleoporin_NSP1 [Alberti, et al. 2009 data]	1/515/8.1e-54/**NS**TFAG; 288/642/7.3e-19/KD; 80/553/2.5e-12/S; 4/280/1.1e-09/T; 63/551/1.5e-08/F; 656/797/2.0e-08/NQ
PD2217	transcriptional_regulatory_protein_SAP30 [Alberti, et al. 2009 data]	25/57/4.6e-30/N

*The biases are in the following format: start point/end point/binomial P-value/bias signature. Residues contributing significantly to the bias are sorted in decreasing order of precedence, *i.e.*, for the bias signature **NS**HIT, N is the main bias, S is the most important subsidiary bias, and so on. NS biases are in bold text. All biases with P-value< = 10^−6^ are listed.

We also assessed the most common of the strongest subsidiary biases that occur in the prionogenic sequences in Table 4 (*i.e.*, the *strongest* biases in each region after the main bias). There are only three patterns that occur more than once: A main N bias, with a strongest subsidiary bias of S occurs 8/25 times; also a Q bias with a strongest subsidiary Y bias occurs twice, as does a Q bias with a strongest subsidiary N bias. These results suggest that experimental investigation into the role of subsidiary serine bias in prion determinants may be informative.

### Relationship to other databases

There are no other published, currently maintained databases for prions and for the wide array of prion-related sequences that are encompassed in PrionHome. Two related published databases do however exist. The Prion Disease Database (PDDB) is a repository of time-course mRNA measurements and other large-scale systems biology data for genes that may change behaviour during prion disease in mammals [71]. Also, AMYPdb, the amyloid precursor database, contains listings of a small subset of prionogenic sequences that have been demonstrated to propagate through amyloid formation [72]. In the future, we will cross-reference our database with these two databases, where possible.

### Conclusions and Future Developments & Updates

We have reported the bioinformatic construction of PrionHome, a comprehensive database resource for prions and other prion-related molecules. To construct the database, we processed the sequences according to a transparent ontology of prionogenic and prion-related protein sequences. We presented some examples of database utility for prion researchers. We used the data in the database to perform compositional analysis of prionogenic proteins in budding yeast, demonstrating prevalent subsidiary compositional bias patterns. As PrionHome develops further, it will become increasingly useful for investigators as a reference database, and also as an aid for further experimental inquiry. Future developments will include the addition of mutation and polymorphism data for all entries in the database, some of which are not deposited in existing standard databases for protein sequence and genetic variation. We are currently performing quality control on the polymorphism data, and it will be added to the second version of the database in the near future. Also, the literature will be curated for new or overlooked reports of interactors of prionogenic proteins. The database will be updated on a weekly basis to incorporate user-submitted data, to add new/overlooked data, and to cover new prions and prion-related phenomena, as they are discovered. More detailed comments, and more comprehensive lists of literature references, will also be added to the database entries, on a regular basis. Links to other databases will be checked regularly for new or changed source data.

As development of this resource is on-going, we will be very happy to receive and act on any constructive comments from peer scientists in the areas of prion biology and protein misfolding, either by email or via the ‘FeedBack’ page on the PrionHome website.

## References

[1] Prusiner SB (1998). Prions.. Proc Natl Acad Sci U S A.

[2] Prusiner SB, Groth D, Serban A, Koehler R, Foster D (1993). Ablation of the prion protein (PrP) gene in mice prevents scrapie and facilitates production of anti-PrP antibodies.. Proc Natl Acad Sci U S A.

[3] Donne DG, Viles JH, Groth D, Mehlhorn I, James TL (1997). Structure of the recombinant full-length hamster prion protein PrP(29–231): the N terminus is highly flexible.. Proc Natl Acad Sci U S A.

[4] Riek R, Hornemann S, Wider G, Billeter M, Glockshuber R (1996). NMR structure of the mouse prion protein domain PrP(121–321).. Nature.

[5] Pan K, Baldwin M, Nguyen J, Gasset M, Serban A (1993). Conversion of alpha-helices into beta-sheets features in the formation of the scrapie prion proteins.. Proc Natl Acad Sci U S A.

[6] Schatzl HM, Da Costa M, Taylor L, Cohen FE, Prusiner SB (1995). Prion protein gene variation among primates.. J Mol Biol.

[7] Wopfner F, Weidenhofer G, Schneider R, von Brunn A, Gilch S (1999). Analysis of 27 mammalian and 9 avian PrPs reveals high conservation of flexible regions of the prion protein.. J Mol Biol.

[8] Goldfarb LG, Brown P, McCombie WR, Goldgaber D, Swergold GD (1991). Transmissible familial Creutzfeldt-Jakob disease associated with five, seven, and eight extra octapeptide coding repeats in the PRNP gene.. Proc Natl Acad Sci U S A.

[9] Zahn R, Liu A, Luhrs T, Riek R, von Schroetter C (2000). NMR solution structure of the human prion protein.. Proc Natl Acad Sci U S A.

[10] Moore R, Lee I, Silverman G, Harrison P, Strome R (1997). Ataxia in prion protein (PrP)-deficient mice is associated with upregulation of the novel PrP-like protein doppel.. J Mol Biol.

[11] Hegde RS, Mastrianni JA, Scott MR, DeFea KA, Tremblay P (1998). A transmembrane form of the prion protein in neurodegenerative disease.. Science (New York, NY.

[12] Watts J, Drisaldi B, Ng V, Yang J, Strome B (2007). The CNS glycoprotein Shadoo has PrP(C)-like protective properties and displays reduced levels in prion infections.. EMBO J e-pub.

[13] Westaway D, Genovesi S, Daude N, Brown R, Lau A (2011). Down-Regulation of Shadoo in Prion Infections Traces a Pre-Clinical Event Inversely Related to PrP Accumulation.. PLoS pathogens.

[14] Rivera-Milla E, Stuermer C, Malaga-Trillo E (2003). An evolutionary basis for scrapie disease: identification of a fish prion mRNA.. Trends Genet.

[15] Harrison PM, Khachane A, Kumar M (2010). Genomic assessment of the evolution of the prion protein gene family in vertebrates.. Genomics.

[16] Schmitt-Ulms G, Ehsani S, Watts JC, Westaway D, Wille H (2009). Evolutionary descent of prion genes from the ZIP family of metal ion transporters.. PloS one.

[17] Lacroute F (1971). Non-Mendelian mutation allowing ureidosuccinic acid uptake in yeast.. J Bacteriol.

[18] Cox B (1965). [PSI], a cytoplasmic suppressor of super-suppression in yeast.. Heredity.

[19] Wickner R (1994). [URE3] as an altered URE2 protein: evidence for a prion analog in Saccharomyces cerevisiae.. Science (New York, NY.

[20] True H, Berlin I, Lindquist S (2004). Epigenetic regulation of translation reveals hidden genetic variation to produce comlex traits.. Nature.

[21] True H, Lindquist S (2000). A yeast prion provides a mechanism for genetic variation and phenotypic diversity.. Nature.

[22] Nakayashiki T, Kurtzman CP, Edskes HK, Wickner RB (2005). Yeast prions [URE3] and [PSI+] are diseases.. Proc Natl Acad Sci U S A.

[23] McGlinchey RP, Kryndushkin D, Wickner RB (2011). Suicidal [PSI+] is a lethal yeast prion.. Proc Natl Acad Sci U S A.

[24] Harrison P, Gerstein M (2003). A method to assess compositional bias in biological sequences and its application to prion-like glutamine/asparagine -rich domains in eukaryotic proteomes.. Genome Biol.

[25] Santoso A, Chien P, Osherovich L, Weissman J (2000). Molecular basis of a yeast prion species barrier.. Cell.

[26] DePace A, Santoso A, Hillner P, Weissman J (1998). A critical role for amino-terminal glutamine/asparagine repeats in the formation and propagation of a yeast prion.. Cell.

[27] Maddelein M, Wickner R (1999). Two Prion-Inducing Regions of Ure2p Are Nonoverlapping.. NMol Cell Biol.

[28] Ross E, Edskes H, Terry M, Wickner R (2005). Primary sequence independence for prion formation.. PNAS.

[29] Ross E, Baxa U, Wickner R (2004). Scrambled prion domains form prions and amyloid.. Mol Cell Biol.

[30] Toombs JA, McCarty BR, Ross ED (2010). Compositional determinants of prion formation in yeast.. Molecular and cellular biology.

[31] Scheibel T, Lindquist SL (2001). The role of conformational flexibility in prion propagation and maintenance for Sup35p.. Nature structural biology.

[32] Pierce MM, Baxa U, Steven AC, Bax A, Wickner RB (2005). Is the prion domain of soluble Ure2p unstructured?. Biochemistry.

[33] Harrison LB, Yu Z, Stajich JE, Dietrich FS, Harrison PM (2007). Evolution of budding yeast prion-determinant sequences across diverse fungi.. J Mol Biol.

[34] Michelitsch MD, Weissman JS (2000). A census of glutamine/asparagine-rich regions: implications for their conserved function and the prediction of novel prions.. Proc Natl Acad Sci U S A.

[35] Si K, Lindquist S, Kandel ER (2003). A neuronal isoform of the aplysia CPEB has prion-like properties.. Cell.

[36] Si K, Choi YB, White-Grindley E, Majumdar A, Kandel ER (2010). Aplysia CPEB can form prion-like multimers in sensory neurons that contribute to long-term facilitation.. Cell.

[37] Gitler AD, Shorter J (2011). RNA-binding proteins with prion-like domains in ALS and FTLD-U.. Prion.

[38] Alberti S, Halfmann R, King O, Kapila A, Lindquist S (2009). A systematic survey identifies prions and illuminates sequence features of prionogenic proteins.. Cell.

[39] Rogoza T, Goginashvili A, Rodionova S, Ivanov M, Viktorovskaya O (2011). Non-Mendelian determinant [ISP+] in yeast is a nuclear-residing prion form of the global transcriptional regulator Sfp1.. Proc Natl Acad Sci USA.

[40] Du Z, Park KW, Yu H, Fan Q, Li L (2008). Newly identified prion linked to the chromatin-remodeling factor Swi1 in Saccharomyces cerevisiae.. Nature genetics.

[41] Brown JC, Lindquist S (2009). A heritable switch in carbon source utilization driven by an unusual yeast prion.. Genes Dev.

[42] Sondheimer N, Lindquist S (2000). Rnq1: An epigenetic modifier of protein function in yeast.. Mol Cell.

[43] Coustou V, Deleu C, Saupe S, Begueret J (1997). The protein product of the het-s heterokaryon incompatibility gene of the fungus Podospora anserina behaves as a prion analog.. Proc Natl Acad Sci U S A.

[44] Patel BK, Gavin-Smyth J, Liebman SW (2009). The yeast global transcriptional co-repressor protein Cyc8 can propagate as a prion.. Nat Cell Biol.

[45] Roberts BT, Wickner RB (2003). Heritable activity: a prion that propagates by covalent autoactivation.. Genes Dev.

[46] Aguzzi A, Rajendran L (2009). The transcellular spread of cytosolic amyloids, prions, and prionoids.. Neuron.

[47] Munch C, O'Brien J, Bertolotti A (2011). Prion-like propagation of mutant superoxide dismutase-1 misfolding in neuronal cells.. Proc Natl Acad Sci U S A.

[48] Sickmeier M, Hamilton JA, LeGall T, Vacic V, Cortese MS (2007). DisProt: the Database of Disordered Proteins.. Nucleic Acids Res.

[49] Hermjakob H, Montecchi-Palazzi L, Lewington C, Mudali S, Kerrien S (2004). IntAct: an open source molecular interaction database.. Nucleic Acids Res.

[50] Altschul SF, Madden TL, Schaffer AA, Zhang J, Zhang Z (1997). Gapped BLAST and PSI-BLAST: a new generation of protein database search programs.. Nucleic Acids Res.

[51] Harrison PM (2006). Exhaustive assignment of compositional bias reveals universally prevalent biased regions: analysis of functional associations in human and Drosophila.. BMC Bioinformatics.

[52] Wickner RB, Edskes HK, Ross ED, Pierce MM, Baxa U (2004). Prion genetics: new rules for a new kind of gene.. Annual review of genetics.

[53] Hou F, Sun L, Zheng H, Skaug B, Jiang QX (2011). MAVS forms functional prion-like aggregates to activate and propagate antiviral innate immune response.. Cell.

[54] Gendoo DM, Harrison PM (2011). Origins and Evolution of the HET-s Prion-Forming Protein: Searching for Other Amyloid-Forming Solenoids.. PloS one.

[55] Harrison P, Zheng D, Zhang Z, Carriero N, Gerstein M (2005). Transcribed processed pseudogenes in the human genome: an intermediate form of expressed retrosequence lacking protein-coding ability.. Nucleic Acids Res.

[56] Harbi D, Kumar M, Harrison PM (2011). LPS-annotate: complete annotation of compositionally biased regions in the protein knowledgebase.. Database (Oxford).

[57] Ward JJ, McGuffin LJ, Bryson K, Buxton BF, Jones DT (2004). The DISOPRED server for the prediction of protein disorder.. Bioinformatics.

[58] Hubbard TJ, Aken BL, Ayling S, Ballester B, Beal K (2009). Ensembl 2009.. Nucleic Acids Res.

[59] Berman H, Henrick K, Nakamura H, Markley JL (2007). The worldwide Protein Data Bank (wwPDB): ensuring a single, uniform archive of PDB data.. Nucleic Acids Res.

[60] Sali A, Blundell TL (1993). Comparative protein modelling by satisfaction of spatial restraints.. J Mol Biol.

[61] Larkin MA, Blackshields G, Brown NP, Chenna R, McGettigan PA (2007). Clustal W and Clustal X version 2.0.. Bioinformatics.

[62] Gould CM, Diella F, Via A, Puntervoll P, Gemund C (2010). ELM: the status of the 2010 eukaryotic linear motif resource.. Nucleic Acids Res.

[63] Tarassov K, Messier V, Landry CR, Radinovic S, Serna Molina MM (2008). An in vivo map of the yeast protein interactome.. Science (New York, NY.

[64] Derkatch I, Bradley M, Zhou P, Chernoff Y, Liebman S (2001). Prions affect the appearance of other prions: The story of [PIN+].. Cell.

[65] Paramithiotis E, Pinard M, Lawton T, LaBoissiere S, Leathers VL (2003). A prion protein epitope selective for the pathologically misfolded conformation.. Nature medicine.

[66] Nelson R, Sawaya M, Balbirnie M, Madsen A, Riekel C (2005). Structure of the cross-beta spine of amyloid-like fibrils.. Nature.

[67] Gazit E (2002). A possible role for pi-stacking in the self-assembly of amyloid fibrils.. FASEB J.

[68] Alexandrov IM, Vishnevskaya AB, Ter-Avanesyan MD, Kushnirov VV (2008). Appearance and propagation of polyglutamine-based amyloids in yeast: tyrosine residues enable polymer fragmentation.. The Journal of biological chemistry.

[69] Ohhashi Y, Ito K, Toyama BH, Weissman JS, Tanaka M (2010). Differences in prion strain conformations result from non-native interactions in a nucleus.. Nature chemical biology.

[70] Halfmann R, Alberti S, Krishnan R, Lyle N, O'Donnell CW (2011). Opposing effects of glutamine and asparagine govern prion formation by intrinsically disordered proteins.. Molecular cell.

[71] Gehlenborg N, Hwang D, Lee IY, Yoo H, Baxter D (2009). The Prion Disease Database: a comprehensive transcriptome resource for systems biology research in prion diseases.. Database (Oxford).

[72] Pawlicki S, Le Bechec A, Delamarche C (2008). AMYPdb: a database dedicated to amyloid precursor proteins.. BMC Bioinformatics.

